# A gene toolbox for monitoring autophagy transcription

**DOI:** 10.1038/s41419-021-04121-9

**Published:** 2021-11-02

**Authors:** Matteo Bordi, Rossella De Cegli, Beatrice Testa, Ralph A. Nixon, Andrea Ballabio, Francesco Cecconi

**Affiliations:** 1 Department of Pediatric Hemato-Oncology and Cell and Gene therapy, IRCCS Bambino Gesù Children’s Hospital, Rome, Italy; 2 Telethon Institute of Genetics and Medicine (TIGEM), Pozzuśoli, Naples Italy; 3 Department of Biology, University of Rome Tor Vergata, Rome, Italy; 4 Center for Dementia Research, Nathan Kline Institute for Psychiatric Research, Orangeburg, NY USA; 5 Departments of Psychiatry, New York University School of Medicine, New York, NY USA; 6 Cell Biology, New York University School of Medicine, New York, NY USA; 7 Department of Molecular and Human Genetics and Neurological Research Institute, Baylor College of Medicine, Houston, TX USA; 8 Medical Genetics Unit, Department of Medical and Translational Science, Federico II University, Naples, Italy; 9 Unit of Cell Stress and Survival, Danish Cancer Society Research Center, Copenhagen, Denmark

**Keywords:** Macroautophagy, Mitophagy

## Abstract

Autophagy is a highly dynamic and multi-step process, regulated by many functional protein units. Here, we have built up a comprehensive and up-to-date annotated gene list for the autophagy pathway, by combining previously published gene lists and the most recent publications in the field. We identified 604 genes and created main categories: MTOR and upstream pathways, autophagy core, autophagy transcription factors, mitophagy, docking and fusion, lysosome and lysosome-related genes. We then classified such genes in sub-groups, based on their functions or on their sub-cellular localization. Moreover, we have curated two shorter sub-lists to predict the extent of autophagy activation and/or lysosomal biogenesis; we next validated the “induction list” by Real-time PCR in cell lines during fasting or MTOR inhibition, identifying ATG14, ATG7, NBR1, ULK1, ULK2, and WDR45, as minimal transcriptional targets. We also demonstrated that our list of autophagy genes can be particularly useful during an effective RNA-sequencing analysis. Thus, we propose our lists as a useful toolbox for performing an informative and functionally-prognostic gene scan of autophagy steps.

## Introduction

Macroautophagy is a pathway of organelle or protein degradation via a typical vesicle, the autophagosome, that promotes recycling of essential cellular components [1]. This process is described as a flux, through which multiple complexes (often coded by autophagy-related genes, ATGs) tightly regulate each one of several sequential steps [2, 3], from an autophagosome nucleation to its fusion with lysosomes and the completion of substrate degradation. Macroautophagy (referred to hereafter as autophagy) can also function selectively, when it promotes degradation of a precise substrate by specific autophagy receptors, such as NDP52, NBR1 and p62/SQSTM1 [2] and by involving the fine coupling of numerous kinases (i.e., AMPK, MTOR, PINK1 and TBK1 among others) [2, 4, 5]. Hence, autophagosomes can also engulf, among other intracellular targets, mitochondria (mitophagy) [6], peroxisomes (pexophagy), ER (ER-phagy), lipid droplets (lipophagy), bacteria (xenophagy) [2], and centriolar satellites (doryphagy) [7]. Moreover, many recent studies discovered novel factors directly or indirectly involved in the regulation of autophagy, with their number constantly growing [8]. Of note, since autophagy activity has been linked to development and progression of a plethora of pathologies [9–11], it becomes increasingly fundamental to develop new strategies for the prediction of a specific autophagy status and/or step [8]. In the last few years, whole transcriptome analysis via RNA-seq technology followed by biological pathway enrichment analysis [12] has been widely used to this end [13, 14]. However, this type of analysis should rely on complete gene lists that accurately described the pathway of interest; to this aim, herein we have curated a comprehensive and up-to-date annotated gene list for autophagy pathway, by combining previously published gene lists [15], most recent publications in the field (reported in the excel file) [8] and bioinformatics tools. Indeed, a handful of autophagy-related resources, available on-line, have been already created collecting diverse data type including functional, structural and biological information: Autophagy, Necrosis, ApopTosis OrchestratorS (THANATOS, http://thanatos.biocuckoo.org) [16], Human Autophagy Database (HADb, http://www.autophagy.lu/), and Human Autophagy Modulator Database (HAMdb, http://hamdb.scbdd.com) [17]. Here, we propose a novel updated gene list that dissects the autophagy pathway and includes the mitophagy process, which can be easily validated experimentally via in silico approaches. Our main objective was to generate an innovative toolbox for an informative and functionally-prognostic gene analysis of all steps of autophagy, by using RNA-seq, that could provide new insights into the molecular mechanisms that drive autophagy regulation or dysfunction. Moreover, starting from the main list of autophagy-related genes, we have curated, as a proof of concept, two shorter sub-lists to predict the two key stages of autophagy activation and lysosomal biogenesis. A so called “activation list” has been derived from the comparison of publications, in which we reported conditions of gene up-regulation upon autophagy activation [18–27]. Also, we validated such activation list by Real-Time (qPCR). The “lysosomal biogenesis list” resulted instead from an extensive literature analysis, obtained by combining data that underwent previous experimental validation [15, 18, 19, 28–30]. Further, we used the “autophagy core” list for gene set enrichment analysis by means of the published RNA-sequencing (RNA-seq) dataset, performed on starved cells [31] or on Down syndrome primary fibroblasts, in which it has been reported an mTORC1 hyperactivation associated to inhibition of autophagy induction [27], and thus demonstrating its effective applicability. Indeed, these two-tier experiments let us conclude that different approaches could be used to generate sub-lists of interests, related to the many steps of autophagy regulation and progression, according to each research need.

## Results

**Fig. 1:**
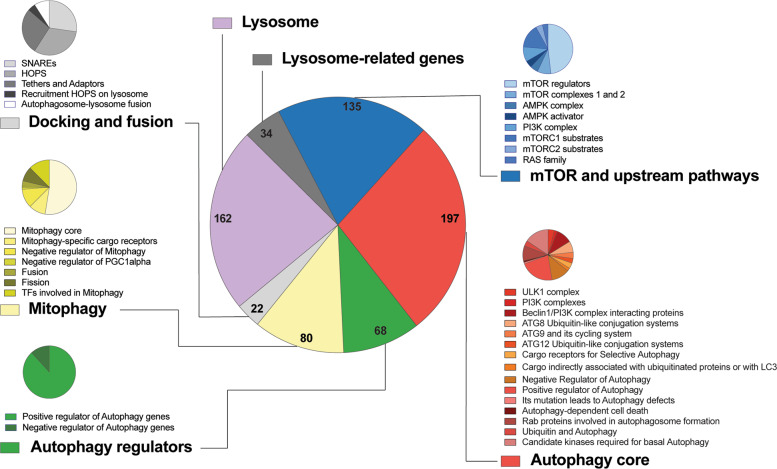
Graphic representation of the 604 genes that compose the entire autophagic list. Genes were classified in 6 main groups: mTOR and upstream pathways (135 genes), autophagy core (197 genes), autophagy regulators (68 genes), mitophagy (80 genes), docking and fusion (22 genes), lysosome (162 genes) and lysosome-related genes (34 genes). For each group, also subgroups are reported.

The 604 genes of our list belong to 6 main categories: MTOR and upstream pathways (135 genes), autophagy core (197 genes), autophagy regulators (68 genes), mitophagy (80 genes), docking and fusion (22 genes), lysosome (162 genes) and lysosome-related genes (34 genes) (Fig. 1, Supplementary Table 1 and Supplementary Figure 1). Within each category, we classified genes in groups based on their functions along the pathway (i.e., ATG8 Ubiquitin-like conjugation systems) or, in other cases, based on their sub-cellular localization (i.e., lysosome) or, lastly, based on the composition of specific complexes (such as MTOR complex 1 or ULK1 complex) (Fig. 1). The lysosome list is based on compartments database (https://compartments.jensenlab.org); moreover, we generated a customized lysosome-related gene list including genes essential for lysosomal activities whose lysosomal location has not yet been experimentally verified. Indeed, some genes belong to more than one category due to their multiple role (indicated in red in the Supplementary Table 1): for example, the regulator of vesicular transport “RAB7A” is located in i) the “HOPS” [32], ii) the “Rab proteins involved in autophagosome formation” [33], and iii) the “mitophagy core” [34] sub-groups. Similarly, the pro-autophagy scaffold factor AMBRA1 is placed in both “Beclin1/PI3K complex interacting proteins” and in “mitophagy core” subgroups. Furthermore, beyond the core ATG proteins critical for autophagosome formation, we classified some genes based on their effect on autophagy, thus creating lists of “Positive regulators of autophagy” and “negative regulators of autophagy” (refer to Supplementary Table 1). Also, we did the same for mitophagy. In addition, we listed mitochondria fission and fusion proteins, as well as specific transcription factors within the mitophagy category; in fact, mitochondrial dynamics and mitophagy are tightly inter-connected and it has been suggested that they regulate each-other [6, 35]. Nonetheless, recent studies demonstrated that mitophagy induction is sustained by the concomitant activation of transcription factors, such as Transcription factor EB (TFEB) and TFE3 [36], that promote lysosomal and autophagic gene expressions. Hence, we performed on the entire list the Gene Ontology Enrichment Analysis (GOEA), by using the DAVID online, to obtain an over-representative analysis of the genes, divided into Biological Process terms, Cellular Compartment terms and Molecular Function terms (Supplementary Figure 2 and Supplementary Table 4).

**Fig. 2:**
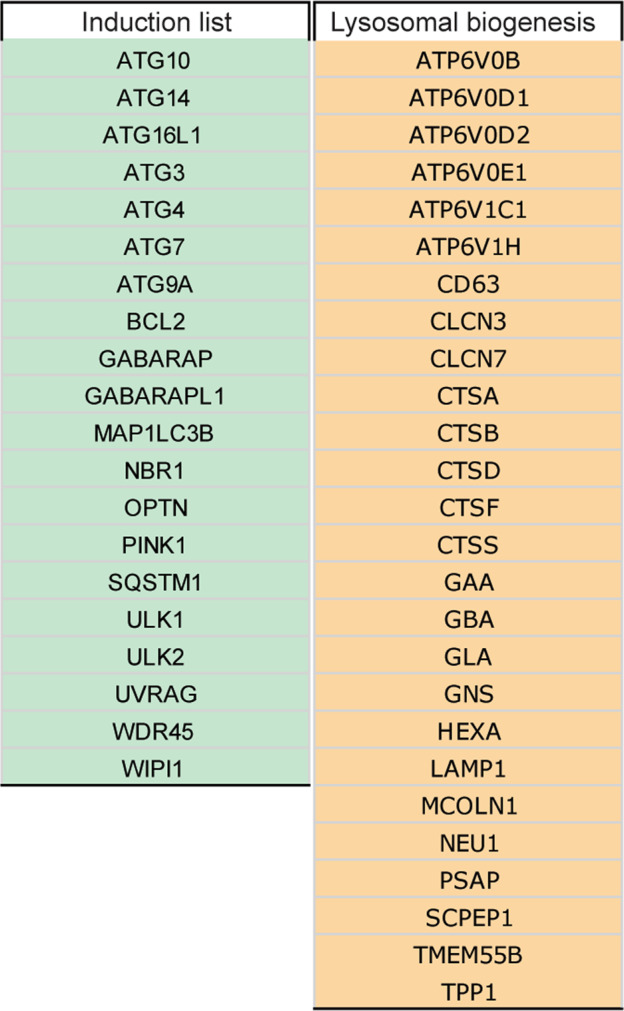
The “induction list” and the “lysosomal biogenesis list”. The two shorter lists were generated by identifying the gene targets related to autophagy activation (Induction list) and lysosomal biogenesis.

**Fig. 3:**
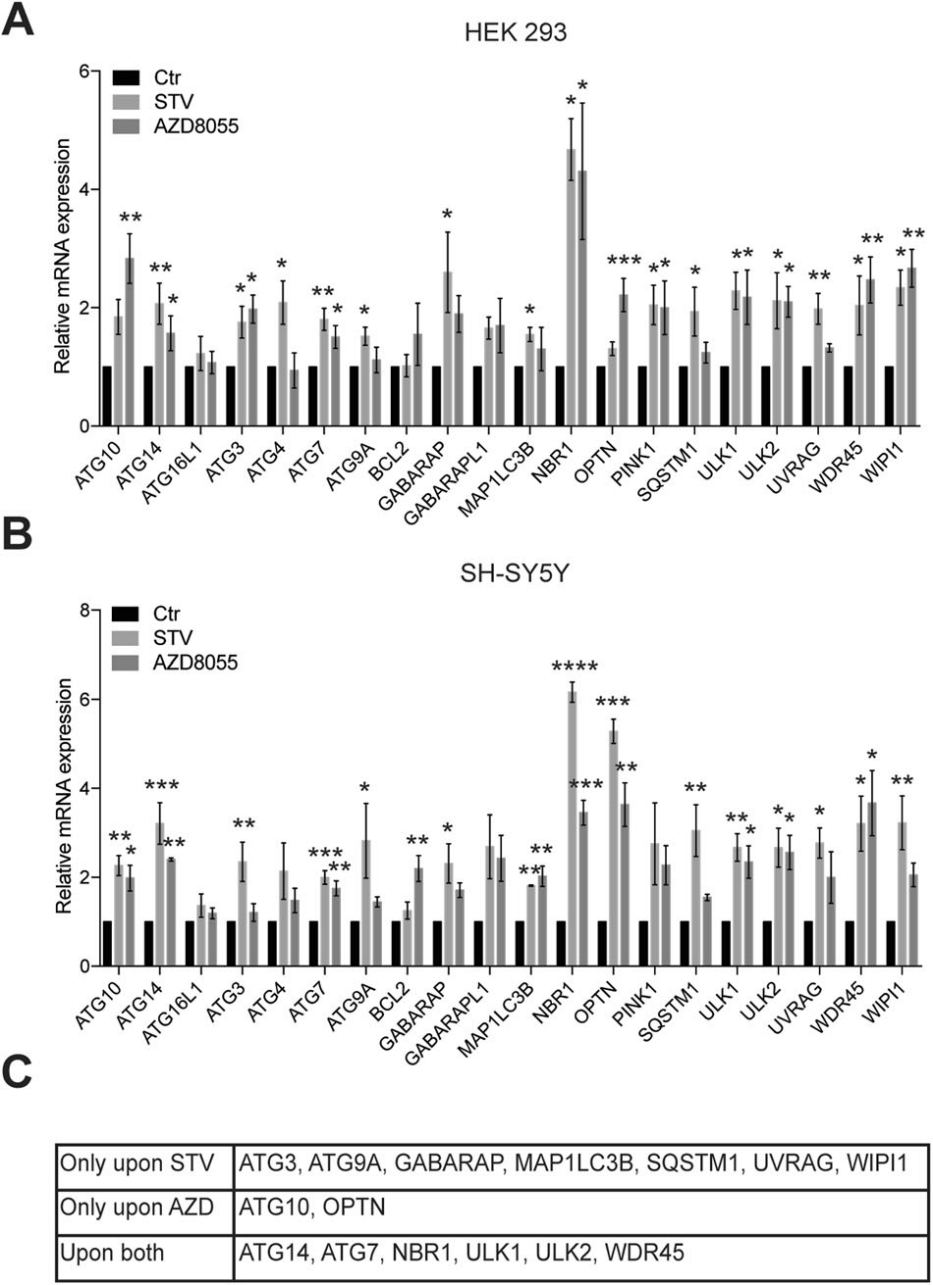
Validation of the “induction list” by Real-time PCR in cell lines during fasting or MTOR inhibition. HEK-293 (**A**) and SH-SY5Y (**B**) cells were cultured in EBSS starvation medium for 4 h or treated with AZD8055 (100 nM) for 8 h. ATG10, ATG14, ATG16L1, ATG3, ATG4, ATG7, ATG9A, BCL2, GABARAP, GABARAPL1, MAP1LC3B, NBR1, OPTN, PINK1, SQSTM1, ULK1, ULK2, UVRAG, WDR45, and WIPI1 mRNA expression were assessed by qPCR and were normalized to HPRT1 mRNA levels, used as internal control. Data display the fold-changes relative to control cells (*n* = 3-4-5-6, based on the variation of each gene expression) and are expressed as the mean value ± SEM. **C** Summary of those genes that were significantly upregulated in both cell lines upon starvation (STV), upon AZD8055 treatment (AZD) or upon both conditions (both). **D** List of genes for the evaluation of lysosomal biogenesis. Statistical analysis was performed using one-way ANOVA with Dunnett’s multiple comparisons test. (*p < 0.05; **p < 0.01; ***p < 0.001; ****p < 0.0001).

Beside the list of 604 genes, we developed as a proof of concept, and by two different approaches two shorter gene lists to predict the activation status of autophagy and the key step of lysosomal biogenesis. In a first case, starting from experimental data available in the literature, we identified 20 targets related to autophagy activation and validated them by qPCR: ATG10, ATG14, ATG16L1, ATG3, ATG4, ATG7, ATG9A, BCL2, GABARAP, GABARAPL1, MAP1LC3B, NBR1, OPTN, PINK1, SQSTM1, ULK1, ULK2, UVRAG, WDR45, WIPI1 (Fig. 2) [18–27]. To this end we used two different cell lines (HEK-293 and SH-SY5Y). We induced autophagy in these systems by (i) classical starvation of amino acids and serum by incubation in EBSS [Earle’s balanced salt solution (EBSS)] for 4 h (HEK-293) and 8 h (SH-SY5Y), or by treating them with AZD8055 (100 nM), a potent mTOR inhibitor [37], for 8 h. Markedly, 4 h or 8 h EBSS treatments are interval times commonly used to induce autophagy; furthermore, we selected 8 h for AZD, since we have previously demonstrated that at this time point a significant elevation in the expression of several autophagy genes [27] can be detected. In order to map a universal “autophagy activation” signature, we next compared the responses of the two cell lines and identified those targets that could have been up-regulated in both conditions: we found that 15 genes out of the 20 were significantly up-regulated upon one or both conditions (Fig. 3A-C). qPCR analysis revealed that mRNA levels of ATG3, ATG9A, GABARAP, MAP1LC3B, SQSTM1, UVRAG and WIPI1 were significantly elevated only upon EBSS; while ATG10 and OPTN were transcriptionally induced only in the presence of AZD. Notably, the expression of ATG14, ATG7, NBR1, ULK1, ULK2 and WDR45 was highly increased by both treatments and in both cell lines (Fig. 3C), indicating that these genes may represent ideal targets for the evaluation of autophagy initiation by transcriptional analyses. Second, it is now clear that an efficient autophagic process requires the concomitant increase in lysosomal activity, for ensuring a proper degradation of autophagic substrates. Hence, for the second sub-lists, we identified 26 targets to assess the state of lysosomal biogenesis (Fig. 2) at variance with the autophagy activation list, this sub-list has been realized by only comparing data from recent work, in which mRNA levels of lysosomal genes in response to autophagy enhancement were accurately and experimentally examined [15, 18, 19, 28–30]. Emerging evidence suggest that modulation of these targets is mostly coordinated by the nuclear translocation of members of the microphthalmia/transcription factor E (MiT/TFE) family that includes TFEB, TFE3, MITF, and TFEC [38].

**Fig. 4:**
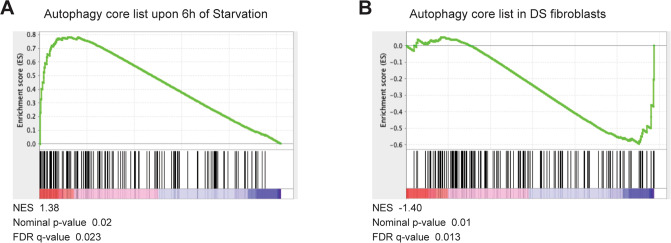
Experimental validation of the “autophagy core” list. GSEA analysis obtained by using the “autophagy core” list, cleaned of “Negative Regulator of Autophagy” and “Its mutation leads to Autophagy defects” sub-categories, in starved HAP1 cells (**A**) or in DS fibroblasts compared to control fibroblast (**B**).

Of note, we have built this comprehensive list of autophagy genes to be particularly useful during an effective RNA-sequencing analysis. Thus, to show its applicability, we performed a gene set enrichment analysis (GSEA) [39, 40] on two previously published RNA-seq datasets, by using the “autophagy core” list: in a first case, RNA-seq was achieved on the human HAP1 cell line, deprived of amino acids and serum for 6 h in EBSS, in which autophagy activation was clearly demonstrated by measuring the autophagic flux [31], and hence represents an excellent positive control. In a second case, the RNA-seq was performed on primary fibroblasts derived from Down Syndrome patients, in which we identified a mitophagy and autophagy deficit associated with mTORC1 hyperactivation [27]; by contrast with the first case, this can therefore be considered as a negative control. The “autophagy core” list was cleaned from the “Negative Regulator of Autophagy” and the “Its mutation leads to Autophagy defects” sub-categories, to better evaluate genes positively regulated upon autophagy induction. Intriguingly, the “autophagy core” list was significantly enriched in starved cells (Normalized Enrichment Score, NES, 1.38, Nominal p-value 0.02, FDR q-value 0.023, Fig. 4A and Supplementary 3A). By contrast, the same list was negatively enriched in DS fibroblasts compared to 2 N control cells (NES -1.40, Nominal p-value 0.01, FDR q-value 0.013, Fig. 4B and Supplementary 3B), thus demonstrating that our list can be easily applied for evaluation of the autophagy state throughout GSEA analysis. Moreover, by analyzing the expression profile of genes that compose the “induction list”, 15 out of 20 were upregulated in starved cells, including ATG14, NBR1, ULK1, and WDR45 (Supplementary Table 2) that we observed to be increased both upon starvation and mTORC inhibition, together with ATG16L1, ATG3, PINK1, BCL2, GABARAPL1, WIPI1, SQSTM1, OPTN, MAP1LC3B, UVRAG, GABARAP. Only DS fibroblasts showed the upregulation of MAP1LC3B, but, on the contrary, 17 targets did not change, and ATG7 and GABARAPL1 were highly downregulated, as previously indicated [27]. Thus, in the presence of mTORC1 activation and consequent autophagy inhibition, these targets are mostly unchanged or downregulated. Next, we evaluated the genes included in the “lysosomal biogenesis” list: in this case, starvation leads to the positive regulation of 13 out of 26 targets (including LAMP1, MCOLN1, and ATP6V0D1), while 3 genes were downregulated, suggesting that -most likely, 6 h starvation may not be sufficient to properly boost the expression of lysosomal genes (Supplementary Table 2), at least in these cells. We earlier reported that DS fibroblasts exhibit a perturbation of lysosomal activities driven by APP-βCTF-dependent compromised luminal acidification [41]; interestingly, in these cells, among the “lysosomal biogenesis” genes, most of them (18) were not changed, while 7 were upregulated and 1 downregulated, CLCN7, which was found to be upregulated under starvation (Supplementary Table 2). Of note, in some instances, such as different cell types or stimuli, we could not rule out the occurrence of an earlier autophagy activation (less than 4 h from a given stimulus). Thus, a time-course experiment was performed to evaluate ATG14, NBR1, ULK1, and WDR45 levels upon starvation or mTOR inhibition in the 30 min-2 h time window. This analysis revealed that these genes showed increased expression at 2 hours only in SH-SY5Y cells, and only upon AZD8055 treatment (Supplementary Figure 4), and thus that short time windows may turn out to be generally insufficient for a robust regulation of gene expression at a transcriptional level.

## Discussion

By our study, we generated a very relevant toolbox for the study of autophagy in a number of interesting conditions, by applying RNA-seq. Nowadays, RNA-seq analysis is increasingly accessible and is largely used for studying biological samples; through the pathway enrichment analysis, researchers can indeed more easily discover or interpret pathways in relation to disease mechanisms or to specific experimental conditions, such as gene KO. Over recent years, the relevance of autophagy and selective autophagy in the context of human pathophysiology has considerably grown. In fact, age- or genetic-related autophagy dysfunction is associated with an extensive number of disorders, such as cancer, diabetes and neurodegenerative diseases [3, 9, 10, 38]. For example, the role of autophagy in cancer cell fate determination is particularly dependent on the cell type or tissue, on the stage of tumor’s development and on the environment (recently reviewed in [42]). Indeed, in this context, it becomes critical to correctly evaluate the potential alteration of a specific autophagy status or step, for understanding whether at all and how autophagy is involved, and then for developing therapies capable of manipulating single mechanisms of autophagy or autophagy regulation. Our comprehensive autophagy transcriptional toolbox is thus highly versatile: it can be easily applied for enrichment analysis by using the entire list or, in alternative, shorter lists can be derived focusing the investigation on selective complexes or functions of the process; Also, one could get useful insights about a putative autophagy status, as demonstrated by re-analyzing published data-sets running the “autophagy core” list. Although the main list is a valuable resource to analyze autophagy in depth, the validated activation-list, made by 20 targets, can also be a very efficient tool. Interestingly, by Real Time PCR upon mTOR inhibition or upon starvation, we identified 6 genes that are fundamental for the proper initiation of the autophagy cascade: ATG14 is a component of the class III PI3K complex 1(PI3KC3-C1), formed by the catalytic subunit vacuolar protein sorting 34 (VPS34), Beclin 1 and general vesicular transport factor p115. This complex activates the phosphatidylinositol-3-phosphate (PI3P) production on the origin site of omegasome [3]. ATG7 is strictly required for autophagosome formation; it is an E1-like activating enzyme present in ubiquitin-like conjugation systems that promotes both cleavage and lipidation of LC3 and GABARAP [43]. NBR1 is an autophagy receptor binding ubiquitylated cargo and recent studies demonstrated that it is also required for selective degradation of peroxisomes (pexophagy) and of ubiquitylated aggregates (aggrephagy) [44]. Intriguingly, NBR1 expression is highly responsive to autophagy activation, more than 3-fold increase compared to control, implying that its promoter is heavily regulated upon nutrient deprivation or mTOR inhibition. ULK1 (Unc-51-like kinase 1) is a Serine/threonine kinase that, together with RB1-inducible coiled-coil protein 1 (FIP200), ATG13, and ATG101 forms the ULK1 complex. Accordingly, ULK1 is considered as the master regulator of autophagy induction, triggering a cascade of signals, through direct phosphorylation of ATG proteins, that culminate in autophagosome formation [3, 43]. Its activation is accurately regulated by mTORC1 and AMPK activity [3]. Furthermore, recent evidence indicates that the ULK2 isoform is a functionally redundant autophagic protein kinase and is ubiquitously expressed such as ULK1 [45]. As ULK1, ULK2 can promote autophagy response following starvation [45], indicating that also this isoform can be informative for the analysis. Obviously, the fact that ULK proteins are kinases, highlights a scenario, in which post-translational modification analysis should integrate in a number of instances, the approach proposed here. Nevertheless, also WDR45 (WD repeat domain 45), that has been found to be important for autophagosome formation, exhibited an excellent positive regulation in the presence of autophagy induction, even though its specific function remains yet unclear [46]. However, other genes, fundamental for the formation and maturation of the autophagosome, i.e., ATG3, ATG9A, GABARAP, MAP1LC3B, UVRAG, and WIPI1 are significantly up-regulated only upon starvation, while mTOR inhibition exerts significant changes in ATG10 and OPTN expression but not on previous gene groups, or at least not in both cell lines. Of note, RNA-seq analysis on starved human HAP1 cells revealed that, besides ATG14, NBR1, ULK1, and WDR45, also ATG16L1, ATG3, PINK1, BCL2, GABARAPL1, WIPI1, SQSTM1, OPTN, MAP1LC3B, UVRAG, GABARAP were significantly upregulated, while upon mTOR hyperactivation, almost all these targets did not show relevant modifications, with the exception of ATG7 and GABARAPL1 (downregulated) or MAP1LC3B (upregulated). Thus, irrespective of the cell type, we may conclude that ATG14, NBR1, ULK1, and WDR45 are high reliable markers for evaluation of autophagy activation, whereas other targets of the induction list may or may not show an adequate modulation in response to autophagy stimulation; this indicates, as it might be expected, the existence of cell-specific regulatory pathways, which in any case do not compromise the reliability of our list. In fact, in DS cells, in which autophagy and mitophagy induction are impaired [27], most of these targets were unchanged or downregulated, with the only exception of MAP1LC3B. Furthermore, during fasting or mTOR inhibition, activation of MiT/TFE transcription factors occurs, with this fostering lysosomal gene expression [47]; again, the appropriate tuning of lysosomal gene expression is strictly related to the duration of the stimuli and to the cell type. In HAP1 cells, after 6 h of starvation, 50% of the genes included in our literature-based lysosome biogenesis list were upregulated, indicating that -most likely, the molecular regulation underlying lysosomal biogenesis requires more prolonged starvation in this cell line. Intriguingly, despite a reduction in lysosomal activity [41], DS cells showed the upregulation of 7 lysosomal targets; this might be explained by the existence of a possible compensatory mechanism or by the effect of other overexpressed genes localized on chromosome 21. Collectively, our research efforts were directed toward developing an inclusive and accurate autophagy transcription gene list functional to autophagy assessment and further identification of autophagy dysfunctions in disease. Thus, discerning the autophagy roles may direct efforts to design therapy strategies based on the adequate modulation of autophagy. Importantly, as we proved by two different approached, additional step-specific sub-lists could be generated by experimental easy approaches or even by digging into solid literature databases, which should include established experimental data.

## Materials and methods

### Generation of the list

The comprehensive autophagic gene list was derived from a broad literature analysis. The list is composed by 646 genes, divided in 6 main categories, as reported in the text. In each category, we created subgroups based on their belonging to the same complex or to a specific regulatory pathway. To this end, we reported at least one reference that has been used to identify the function of each single gene. The references are indicated in the column A of the excel file; the column B of the file states the Biological Function, while the Column C, D, and E display the official gene symbol, the common name of the related protein (alias), and the description, respectively. Since some genes are present in more than one group, due to their multiple functions along the autophagy process, these genes are listed more than once and are marked with the red color. The restricted “induction list” for the prediction of autophagy activation state, made by 20 targets, was obtained combining data from previous publications [18–27], in which have been documented the increase of the mRNA expression following autophagy induction. Similarly, the list for the evaluation of “lysosomal biogenesis” was created from literature by combining data that underwent preceding experimental validation [15, 18, 19, 28–30].

### Cell culture and treatments

The human embryonic kidney HEK293 cell line were cultured in Dulbecco’s modified Eagle’s medium (DMEM, Euroclone), while human neuroblastoma SHSY-5Y cell line in DMEM-F12 (1:1, Gibco) at 37 °C under 5% CO_2_; both mediums were supplemented with 10% heat inactivated fetal bovine serum (FBS, Gibco), 2mM L-glutamine, 100 U/ml penicillin and 100 mg/ml streptomycin (Gibco). The induction of autophagy by nutrients starvation was obtained by incubating HEK293 cells with Earle’s balanced salt solution (EBSS; Sigma-Aldrich) for 4 h and SHSY-5Y cells for 8 h. In the other case, autophagy stimulation was obtained by treating cells with 100 nM AZD8055 (AZD), an ATP-competitive inhibitor directly targeting the mTOR catalytic site (Selleckchem) [37], for 8 h. Both cells lines were also starved or treated with AZD8055 for 30 min, 1 h, 1.5 h, and 2 h, as indicated in figure legend.

### Preparation of cDNA and Real-Time (qPCR)

RNA was extracted from cells using ReliaPrep™ RNA Cell Miniprep System (Promega) according to the manufacturer’s specifications, including DNase treatment step. RNA quantity was determined with a Nanodrop 1000 spectrophotometer (Wilmington, DE). cDNA was prepared from total RNA using GoScript™ Reverse Transcription Mix according to manufacturer’s instructions and using Random Primers (Promega). Following reverse transcription, qPCR was then performed using SYBR Green Mix (Thermo Fisher Scientific) with the STEPONE Real-Time PCR System (Applied Biosystems™). The oligos primers are listed in Supplementary Table 3.

The target genes were normalized against HPRT1 that was used as a housekeeping gene. Results were calculated using the ΔΔCt method. We then normalized the mRNA levels to the control condition. Reported values are the means of at least three independent biological replicates with technical triplicates that were averaged for each experiment. Error bars represent the SEM of the mean.

### Gene set and ontology enrichment analysis

Gene set enrichment analyses were performed according to standard procedures [39, 40] on two published RNA-seq datasets [31, 48]; GSEA was run by using the “autophagy core” list from Supplementary Table 1, cleaned of “Negative Regulator of Autophagy” and “Its mutation leads to Autophagy defects” sub-categories. The Gene Ontology Enrichment Analysis (GOEA) [49, 50] was performed by using the DAVID online tool (DAVID Bioinformatics Resources 6.8) [51] restricting the output to Biological Process terms (BP_FAT), Cellular Compartment terms (CC_FAT), and Molecular Function terms (MF_FAT). GOEA results are reported in Supplementary Table 4. In Supplementary Figure 3, we plotted the top 10 BP, CC and MF significantly enriched terms. The threshold for statistical significance of GOEA was FDR < 0,1 and the Enrichment Score≥1.5. Heatmaps were generated using GraphPad Prism program (version 7), showing the log2fold change of each gene.

### Statistical analysis

Statistical significance was measured using one-way ANOVA with Dunnett’s multiple comparisons test calculated with GraphPad Prism program (version 7), as reported in the figure legend.

## Supplementary information

Table 1

Table 2

Supplementary Table 3

Supplementary Figure 1

Supplementary Figure 2

Supplementary Figure 3

Supplementary Figure 4

Supplementary Table 4
