# Supplementary figures and images for: A gene toolbox for monitoring autophagy transcription
Source: Cell Death Dis. 2021 Nov 2;12(11):1044. doi: 10.1038/s41419-021-04121-9 (PMC8563709; doi:10.1038/s41419-021-04121-9)

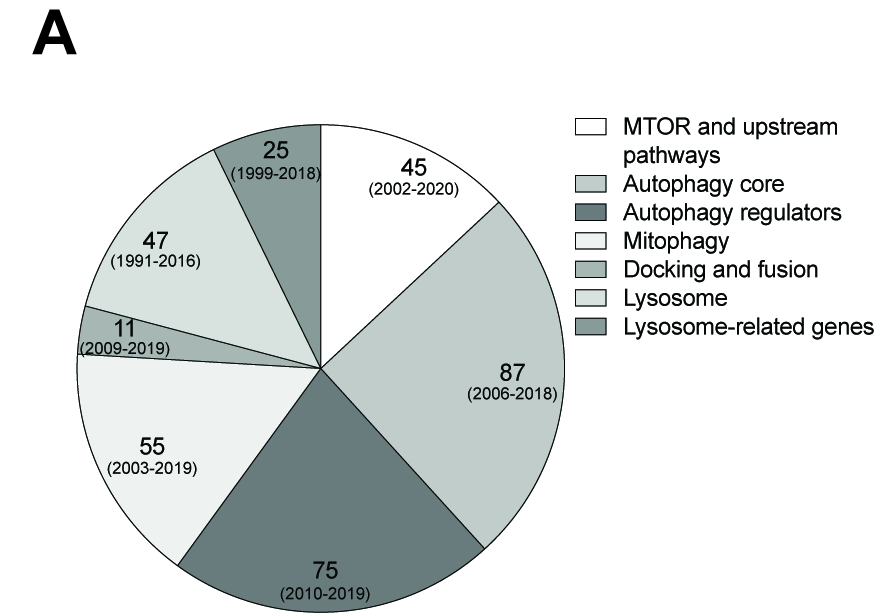

Supplement: Supplementary file 4 — Supplementary Figure 1 [file 41419_2021_4121_MOESM4_ESM.tif]

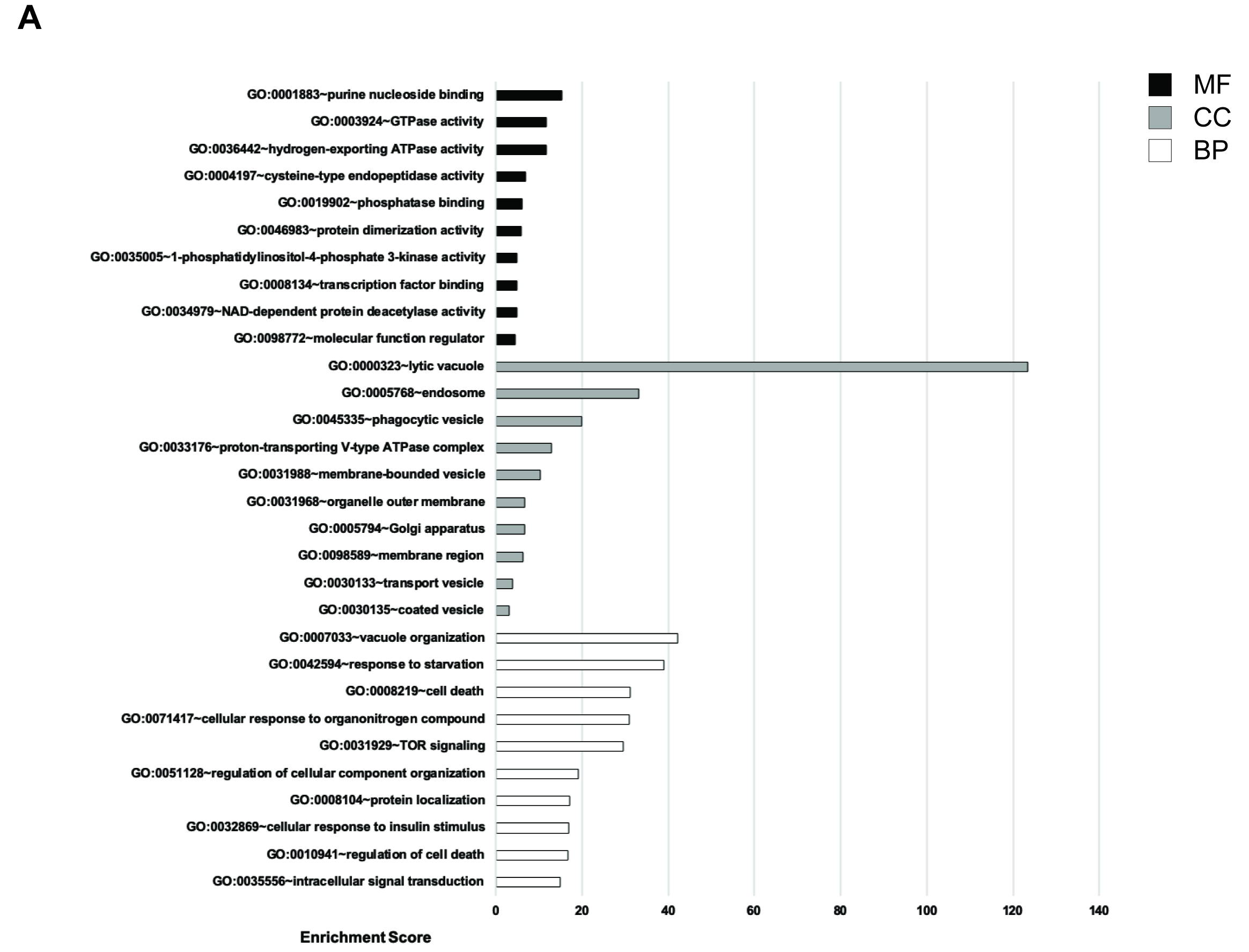

Supplement: Supplementary file 5 — Supplementary Figure 2 [file 41419_2021_4121_MOESM5_ESM.tif]

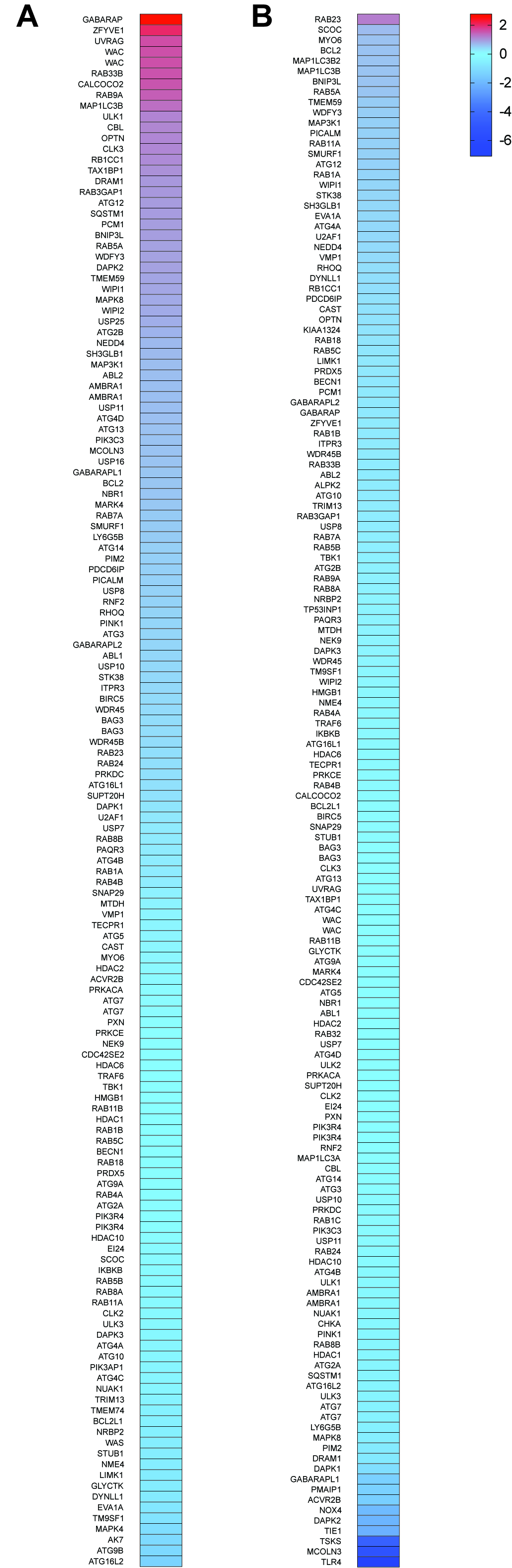

Supplement: Supplementary file 6 — Supplementary Figure 3 [file 41419_2021_4121_MOESM6_ESM.tif]

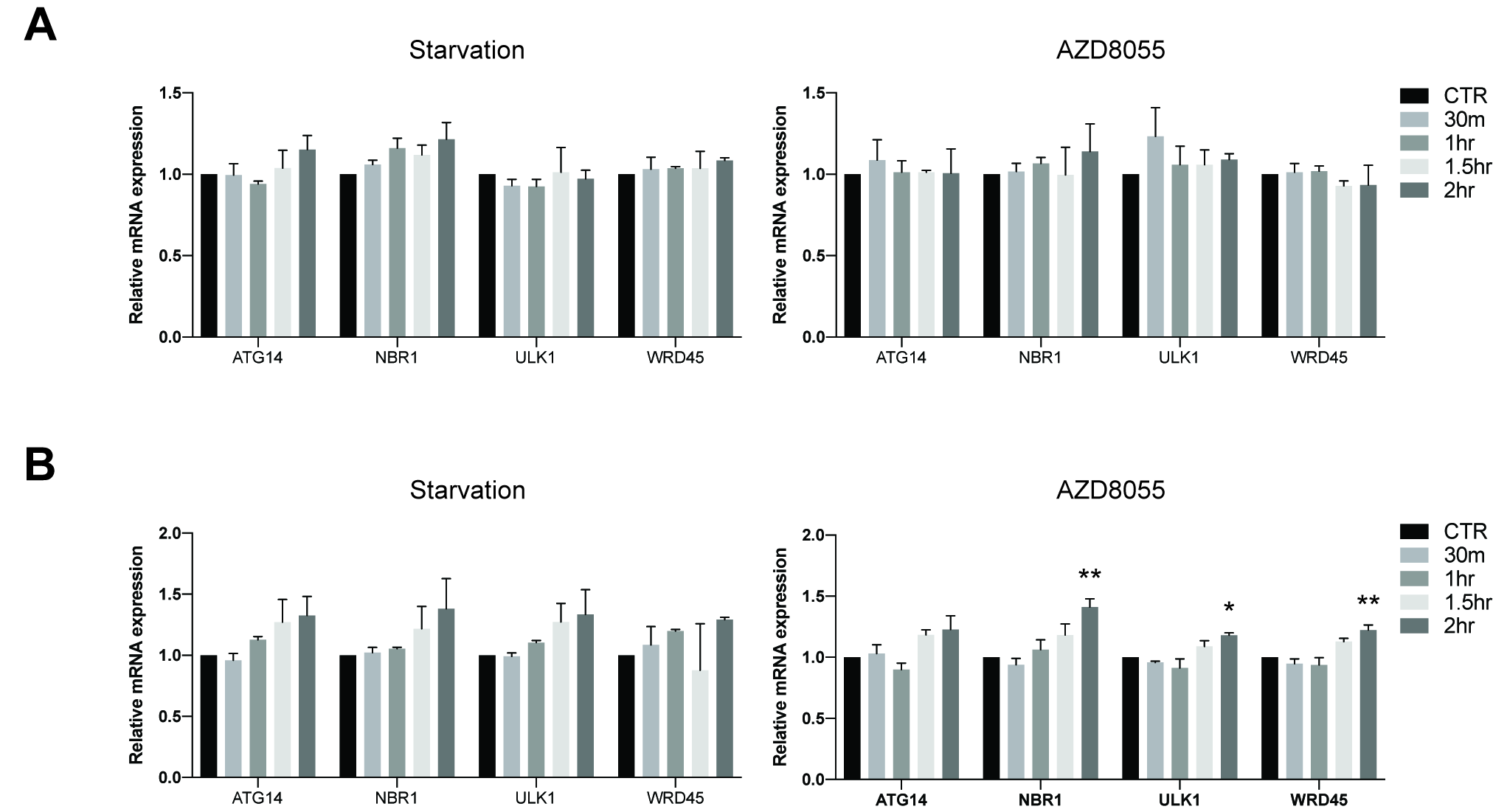

Supplement: Supplementary file 7 — Supplementary Figure 4 [file 41419_2021_4121_MOESM7_ESM.tif]
